# Characterizing trends and patterns in benzodiazepine-related toxicity deaths in Ontario: A descriptive cross-sectional study

**DOI:** 10.1371/journal.pone.0355210

**Published:** 2026-09-15

**Authors:** Tonya Campbell, Shaleesa Ledlie, Pamela Leece, Joanna Yang, Bisola Hamzat, Gillian Kolla, Nikki Bozinoff, Rob Boyd, Mike Franklyn, Ashley Smoke, Dana Shearer, Tara Gomes

**Affiliations:** 1 MAP Centre for Urban Health Solutions, St. Michael’s Hospital, Toronto, Ontario, Canada; 2 Li Ka Shing Knowledge Institute, St. Michael’s Hospital, Toronto, Ontario, Canada; 3 Epidemiology Research Unit, Caribbean Institute for Health Research, The University of the West Indies, Mona, Kingston, Jamaica; 4 Public Health Ontario, Toronto, ON, Canada; 5 Dalla Lana School of Public Health, University of Toronto, Toronto, ON, Canada; 6 Department of Family and Community Medicine, University of Toronto, Toronto, ON, Canada; 7 ICES, Toronto, Ontario, Canada; 8 Faculty of Medicine, Memorial University, St. John’s, NL, Canada; 9 Campbell Family Mental Health Research Institute, Centre for Addiction and Mental Health, Toronto, ON, Canada; 10 Ottawa Inner City Health Inc., Ottawa, ON, Canada; 11 Northern Ontario School of Medicine, Sudbury, ON, Canada; 12 Ontario Drug Policy Research Network Lived Experience Advisory Group, Toronto, ON, Canada; 13 Leslie Dan Faculty of Pharmacy, University of Toronto, Toronto, ON, Canada; 14 Institute of Health Policy, Management and Evaluation, University of Toronto, Toronto, ON, Canada; Tekirdag Namik Kemal University: Tekirdag Namik Kemal Universitesi, TÜRKIYE

## Abstract

**Objectives:**

The unregulated drug supply in Canada increasingly contains benzodiazepines, particularly those not approved for medical use, raising concerns about toxicity risk, especially when combined with opioids. Therefore, we aimed to describe trends in benzodiazepine-related toxicity deaths and compare their characteristics and circumstances by benzodiazepine type.

**Methods:**

We conducted a descriptive cross-sectional study of accidental benzodiazepine-related toxicity deaths in Ontario between January 1, 2018 and June 30, 2022, using linked administrative healthcare at ICES including coroner’s investigation records from the Drug and Drug/Alcohol Related Death Database. We measured monthly crude rates of benzodiazepine toxicity deaths and summarized characteristics (age, sex, region of residence, etc.) and circumstances (number and types of substances involved, healthcare encounters for toxicities and substance use disorders, etc.) of decedents and compared these by benzodiazepine type (approved vs. not approved for medical use in Canada).

**Results:**

Between January 2018 and June 2022, 782 Ontarians died of benzodiazepine-related toxicity (median age of 40 years), of which 70.5% were male and 88.6% resided in Southern Ontario The rate of benzodiazepine toxicity death peaked in December 2020 (0.27 per 100,000; N = 40). Overall, 98.1% of benzodiazepine deaths involved another substance, most commonly opioids (91.2%). Moreover, one-fifth (22.6%) of decedents had experienced a hospital-treated substance toxicity in the year prior to death, and almost two-thirds (64.1%) had a healthcare encounter with a substance use disorder diagnosis in the prior five years.

**Conclusion:**

Benzodiazepines remain an important contributor to substance-related mortality in Ontario, with nearly 800 benzodiazepine-related toxicity deaths observed over a four-and-a-half-year period. As almost all deaths involved multiple substances, most commonly opioids, harm reduction and treatment programs must adapt to the evolving needs of people who use drugs who may experience both intentional and unintentional benzodiazepine exposure.

## Introduction

The emergence of novel benzodiazepines in the unregulated drug supply is a rising public health concern across Canada and the US [1–3] as well as globally [4]. Increasingly, the unregulated opioid supply across Canada has been contaminated with benzodiazepines, [5] including those not approved for medical use (often-termed ‘designer’ benzodiazepines such as etizolam and flualprazolam) [4,6] as well as traditional benzodiazepines. Regardless of type, benzodiazepines may also be bought for intentional use on the unregulated market. Although the risk of acute toxicity from the consumption of benzodiazepines alone is relatively low, their increasing presence in the unregulated drug supply alongside fentanyl is particularly concerning, as the risk of benzodiazepine-related toxicity is greatly heightened when combined with other central nervous system depressants, such as opioids [7,8]. Between 2018 and 2023, the rate of benzodiazepine toxicity deaths in Canada increased almost four-fold, from 1.2 to 5.6 deaths per 100,000 [9,10]. Much of this increase was observed among young adults, alongside substantial declines among individuals without an active benzodiazepine prescription [11]. In 2023 and 2024, approximately half of all fentanyl-containing drug samples analysed by Health Canada’s Drug Analysis Service also contained a benzodiazepine not approved for use in Canada [12]. Moreover, the effects of these novel benzodiazepines, as well as the short- and long-term risks associated with their use, have not been well-studied, driving concerns about complications in toxicity response, and an increasing prevalence of benzodiazepine-related dependence and associated withdrawal.

As the unregulated drug supply continues to shift, with an increasing prevalence of benzodiazepines not approved for medical use in Canada, there is an urgent need to understand how these dynamics are affecting acute benzodiazepine-related outcomes, including trends over time and circumstances surrounding death. These data are necessary to identify opportunities for improving existing harm reduction initiatives and developing additional strategies to address the evolving drug toxicity crisis. Accordingly, our objective was to describe contemporary trends in benzodiazepine-related toxicity deaths in Ontario and compare the characteristics and circumstances of death by benzodiazepine type.

## Methods

### Study design and setting

We conducted a population-based, cross-sectional study of all accidental benzodiazepine-related toxicity deaths that occurred in Ontario, Canada between January 1, 2018 and June 30, 2022. Accidental benzodiazepine toxicity deaths were defined as those arising from an acute intoxication due to the direct contribution of a benzodiazepine, either alone or in combination with other drugs or substances, and which were confirmed as unintentional (i.e., an injury where death was not intended, foreseen or expected) by the investigating coroner. The study is reported as per the Strengthening the Reporting of Observational Studies in Epidemiology (STROBE) guidelines [13] with a preliminary analysis of this data previously made available online [14,15].

### Data sources

We obtained data from ICES (formerly known as the Institute for Clinical Evaluative Sciences), an independent, non-profit research institute with legal status allowing for the analysis of administrative data for healthcare system evaluation and improvement. To identify people who died of benzodiazepine-related toxicity, we used the Drug and Drug/Alcohol Related Death Database, which holds records from coroner’s investigations of all confirmed toxicity deaths where benzodiazepines, opioids, alcohol, and/or stimulants directly contributed to death in Ontario. The database captures post-mortem toxicology of the substances directly involved and contributing to death, as well as sociodemographic information and details about the manner and circumstances surrounding death as determined by the investigating coroner, including the location of the acute toxicity incident, the number and type of substances contributing to death, and whether a bystander was present.

To enumerate the population of Ontario and ascertain the sociodemographic characteristics of the study population, we used the Registered Persons Database, a registry of all Ontario residents eligible for the universal and publicly funded Ontario Health Insurance Plan. We used the Narcotics Monitoring System, which contains all prescriptions for controlled substances dispensed from community pharmacies in Ontario regardless of the payer, to capture prior dispensed prescriptions for benzodiazepines and opioids. We identified healthcare encounters in the emergency department and in inpatient settings using the Canadian Institute for Health Information’s (CIHI) National Ambulatory Care Reporting System and Discharge Abstract Database, respectively, and we ascertained visits to outpatient care using the Ontario Health Insurance Plan Claims Database. These datasets were linked using unique encoded identifiers and analyzed at ICES. The use of the data in this project is authorized under section 45 of Ontario’s Personal Health Information Protection Act and does not require review by a Research Ethics Board. The study team did not have access to any identifying personal information and accessed the data for research purposes between September 29, 2023 and January 31, 2025.

### Measures

We measured the monthly rate of benzodiazepine-related toxicity deaths to examine temporal trends over the study period. We summarized the characteristics of each person who died of benzodiazepine-related toxicity over the study period and the circumstances surrounding their death. This included age (0–24, 25–44, 45–64, 65 + years), sex, neighbourhood income quintile (using CIHI’s standard definition [16]), location of residence (urban or rural region; northern or southern region), setting of the toxicity incident (private residence, other, unknown), other substances (opioids, alcohol, or stimulants) directly contributing to death, and the number substances directly involved in the death (benzodiazepines alone or multiple substance). We also assessed the prevalence of dispensed prescriptions for benzodiazepines and opioids in the 30 days, one year, and five years prior to death. Finally, we summarized prior healthcare encounters for acute substance-related toxicities (due to benzodiazepines, opioids, alcohol, or stimulants) in the year before death, and for substance use disorder diagnoses (any substance, and benzodiazepines specifically) in the five years before death.

We stratified all characteristics and circumstances according to the type of the benzodiazepine (approved vs. not-approved for medical use) directly contributing to death, based on each benzodiazepines’ status in Canada (i.e., whether each benzodiazepine is legally available by prescription) [17]. Benzodiazepines not approved for medical use in Canada included: etizolam, flubromazolam, flualprazolam, and bromazolam, while benzodiazepines approved for medical use in Canada included: alprazolam, bromazepam, camazepam, chlordiazepoxide, clobazam, clonazepam, demoxepam, diazepam, flurazepam, hydroxyalprazolam, lorazepam, midazolam, nitrazepam, oxazepam, temazepam, and clorazepate. Deaths were attributed to non-approved benzodiazepines if post mortem toxicology identified these substances, either alone or in combination with an approved benzodiazepine. Deaths were attributed to an approved benzodiazepine if toxicology identified only benzodiazepines approved for medical use. See S1 Table for details on definitions, diagnosis codes and benzodiazepine types.

### Statistical analysis

We calculated the monthly crude rate of benzodiazepine-related toxicity deaths (per 100,000 Ontario residents) across the study period. We summarized the characteristics and circumstances of people who died of benzodiazepine-related toxicity using descriptive statistics, reported overall and stratified by the type of benzodiazepine directly involved in death. We used chi-square tests and Fisher’s exact tests to compare characteristics and circumstances according to benzodiazepine type. All analyses were conducted at ICES using SAS Enterprise Guide 8.3 (SAS institute, Cary, North Carolina, USA), and used a type 1 error rate of 0.05 as the threshold for statistical significance.

### Involvement of people with lived experience

The Ontario Drug Policy Research Network (ODPRN) regularly engages a Lived Experience Advisory Group, comprised of people with lived and living experience using opioids. Throughout the study, we consulted with this group, along with a separate group of five individuals with lived and living experience using a range of substances, including opioids, stimulants, benzodiazepines, and alcohol, about the study scope, methodology, and interpretation of the results. Throughout the conduct of this work, we met six times, where these individuals provided feedback on the study scope, methodology, contextualization, and reporting of results. All people with lived and living experience were compensated for their time spent on this study in line with standard best practices, [18] and were offered co-authorship (or when preferred by the individual, acknowledgment) in this manuscript.

## Results

### Trends

**Fig 1:**
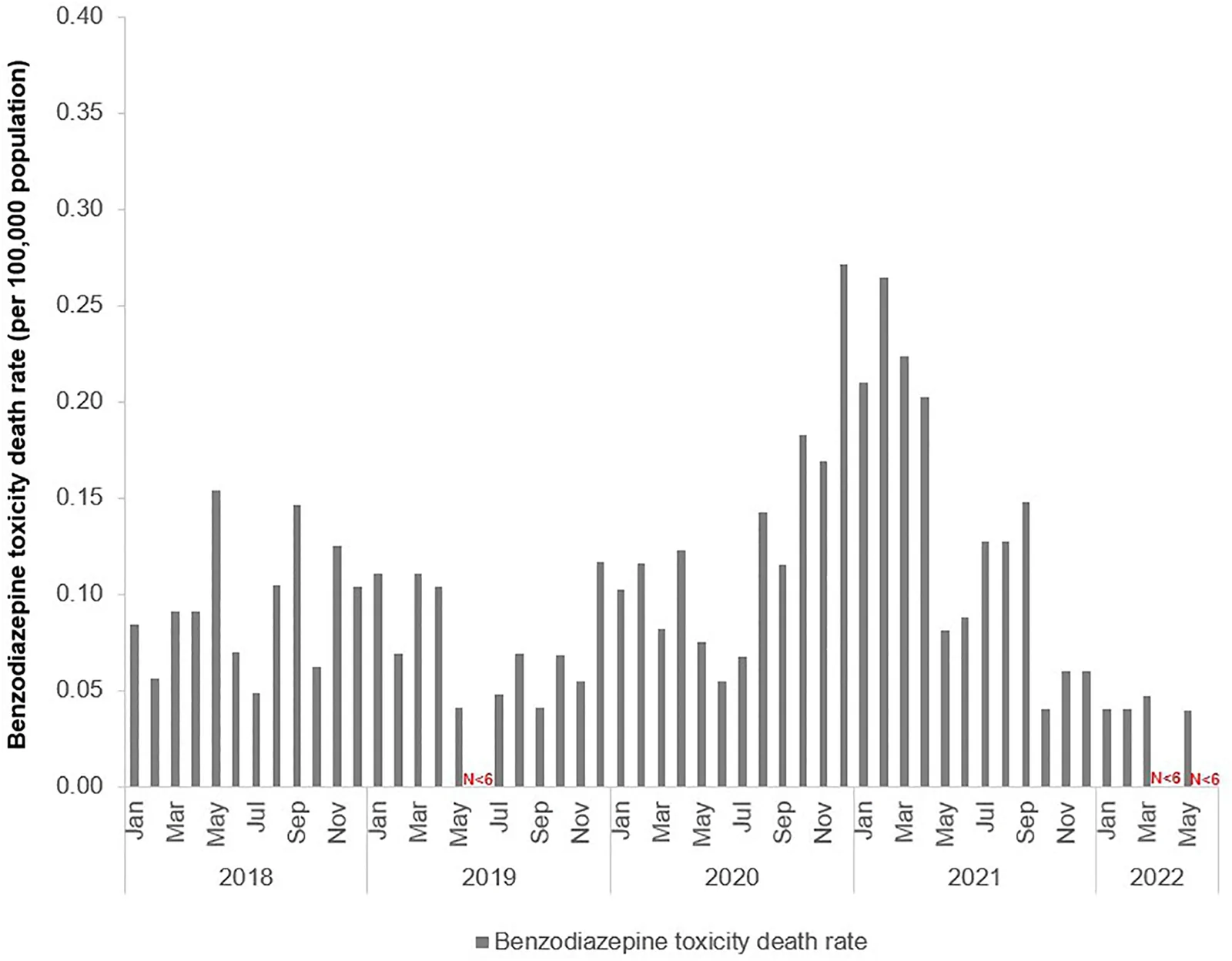
Trends in the rate of benzodiazepine-involved toxicity deaths in Ontario, January 2018 to June 2022.

Between January 2018 and June 2022, 782 Ontarians had an accidental death involving benzodiazepine toxicity. Over the first two-and-a-half years of the study period, the crude rate of benzodiazepine-related toxicity death remained relatively stable (from 0.08 per 100,000 in January 2018 [N = 12] to 0.05 per 100,000 in June 2020 [N = 8]), notwithstanding minor seasonal variations in rates observable as troughs in summer months and peaks during cooler months (Fig 1). However, in July 2020, rates of benzodiazepine-related toxicity death began to rise in Ontario, reaching a peak of 0.27 per 100,000 (N = 40) in December 2020. By October 2021, the rate of benzodiazepine-related toxicity deaths had declined to 0.04 deaths per 100,000 (N = 6) and remained relatively stable for the remainder of the study period (Fig 1).

### Characteristics and circumstances

Over the study period, benzodiazepine-related toxicity deaths were evenly distributed by benzodiazepine type, with half (50.1%; N = 392) involving benzodiazepines approved for medical use, and the other half (49.9%; N = 390) involving benzodiazepines not approved for medical use (Table 1). Overall, the median age at death was 40 years (interquartile range [IQR] 30–53 years), although this was higher for deaths related to approved benzodiazepines (median age: 45 years [IQR: 33–56 years] vs. 37 years [IQR: 28–49 years] for deaths attributed to non-approved benzodiazepines; p < 0.001). Males accounted for more than two-thirds of deaths (70.5%; N = 551), and this predominance was more pronounced for deaths involving benzodiazepines not approved vs. approved for medical use (76.9% vs. 64.0%; p < 0.001). Overall, most benzodiazepine-related toxicity deaths occurred among people residing in urban (91.2%; N = 713) and southern (88.6%; N = 693) regions of the province. Notably, northern-residing individuals accounted for a larger proportion of deaths with benzodiazepines approved vs. not approved for medical use (14.8% vs. 7.9%; p = 0.003). In general, deaths were concentrated in lower-income neighbourhoods, with approximately 60% of decedents (N = 462; 59.1%) residing in communities in the two lowest quintiles of income (Table 1).

**Table 1:** Characteristics of Ontarians who died of benzodiazepine-involved toxicity, January 1, 2018 to June 30 2022.

	Overall	Benzodiazepines – not approved for medical use	Benzodiazepines -approved for medical use	*p*-value*
**Number of individuals**	782	390	392	
**Age, years**				
Median (IQR)	40 (30-53)	37 (28-49)	45 (33-56)	<0.001
0-24	89 (11.4%)	58−62 (14.9%−15.9%)	27−31 (6.9%−7.9%)	
25-44	373 (47.7%)	209 (53.6%)	164 (41.8%)	
45-64	291 (37.2%)	118 (30.3%)	173 (44.1%)	
65+	29 (3.7%)	1−5 (0.3%−1.3%)	24−28 (6.1%−7.1%)	
**Sex**				<0.001
Female	231 (29.5%)	90 (23.1%)	141 (36.0%)	
Male	551 (70.5%)	300 (76.9%)	251 (64.0%)	
**Income quintile of neighbourhood of residence**				0.647
1 (lowest income)	302 (38.6%)	147 (37.7%)	155 (39.5%)	
2	160 (20.5%)	78 (20.0%)	82 (20.9%)	
3	128 (16.4%)	69 (17.7%)	59 (15.1%)	
4	87 (11.1%)	40 (10.3%)	47 (12.0%)	
5 (highest income)	95 (12.1%)	49 (12.6%)	46 (11.7%)	
Unknown	10 (1.3%)	7 (1.8%)	3 (0.8%)	
**Rurality of region of residence**				0.297
Urban	713 (91.2%)	357 (91.5%)	356 (90.8%)	
Rural	59 (7.5%)	26 (6.7%)	33 (8.4%)	
Unknown	10 (1.3%)	7 (1.8%)	3 (0.8%)	
**Residence in northern or southern Ontario**				0.003
Northern	89 (11.4%)	31 (7.9%)	58 (14.8%)	
Southern	693 (88.6%)	359 (92.1%)	334 (85.2%)	

**Note:** Data are presented as median (interquartile range) for continuous variables, and no. (%) for categorical variables.

Boldface *p*-value indicates statistically significant difference (α = 0.05) when comparing deaths attributed to approved vs. non-approved benzodiazepines.

Three-quarters (75.4%; N = 590) of all benzodiazepine-related toxicity deaths occurred at a private residence, and this was even more common among those involving benzodiazepines approved for medical use (79.8% vs. 71.0%; p = 0.007; Table 2). Moreover, the vast majority of deaths (98.1%; N = 767) involved more than one substance. Opioids were the most common co-occurring substance, with direct involvement in 9 of 10 benzodiazepine-related toxicity deaths overall (91.2%; N = 713), more commonly among deaths involving benzodiazepines not approved for medical use (95.1% vs. 87.2%; p < 0.001). Stimulants were co-involved in approximately half of all benzodiazepine-related toxicity deaths (47.1%; N = 368); however, this prevalence was considerably higher among deaths involving benzodiazepines not approved for medical use (60.5% vs. 33.7%; p < 0.001). Alcohol was involved in 18.5% of all benzodiazepine-related toxicity deaths (N = 145), and this was more common among deaths involving approved benzodiazepines (24.5% vs. 12.6%; p < 0.001), in contrast to the pattern observed with stimulants. With regards to prescribing history, we observed a much higher prevalence of benzodiazepine dispensing among deaths attributed to benzodiazepines approved for medical use, in the 30 days (60.7% vs. 10.8%; p < 0.001), one year (70.4% vs. 20.8%; p < 0.001), and five years before death (80.1% vs. 36.9%; p < 0.001). A similar pattern was observed for a history of opioid prescribing in the 30 days (46.7% vs. 24.9%; p < 0.001), one year (66.8% vs. 46.9%; p < 0.001), and five years prior to death (85.5% vs. 73.3%; p < 0.001). Notably, one-fifth (22.6%; N = 177) of all Ontarians who died of benzodiazepine-related toxicity experienced a hospital-treated substance toxicity in the year prior to death. This history was more common among deaths involving benzodiazepines not approved for medical use (27.4% vs. 17.9%; p = 0.001), driven by a history of opioid toxicity in particular (25.1% vs. 13.8%; p < 0.001). Finally, in the five years preceding death, nearly two-thirds (64.1%; N = 501) of decedents had a healthcare encounter where a substance use disorder diagnosis was recorded, and this was similar across benzodiazepine type (Table 2).

**Table 2:** Circumstances surrounding and preceding death among people who died of benzodiazepine-involved toxicity, January 1, 2018 to June 30 2022.

	Overall	Benzodiazepines – not approved for medical use	Benzodiazepines -approved for medical use	*p*-value*
**Number of individuals**	782	390	392	
**Location of toxicity incident**				0.007
Private Residence	590 (75.4%)	277 (71.0%)	313 (79.8%)	
Other^†^	186 (23.8%)	111 (28.5%)	75 (19.1%)	
Unknown	6 (0.8%)	2 (0.5%)	4 (1.0%)	
**Number of substances involved in death**				0.44
1 (monosubstance)	15 (1.9%)	6 (1.5%)	9 (2.3%)	
2+ (polysubstance)	767 (98.1%)	384 (98.5%)	383 (97.7%)	
**Other substances directly contributing to death**				
Opioids	713 (91.2%)	371 (95.1%)	342 (87.2%)	<0.001
Alcohol	145 (18.5%)	49 (12.6%)	96 (24.5%)	<0.001
Stimulants	368 (47.1%)	236 (60.5%)	132 (33.7%)	<0.001
**Prior dispensing of prescription benzodiazepines**				
30 days	280 (35.8%)	42 (10.8%)	238 (60.7%)	<0.001
1 year	357 (45.7%)	81 (20.8%)	276 (70.4%)	<0.001
5 years	458 (58.6%)	144 (36.9%)	314 (80.1%)	<0.001
**Prior dispensing of prescription opioids**				
30 days	280 (35.8%)	97 (24.9%)	183 (46.7%)	<0.001
1 year	445 (56.9%)	183 (46.9%)	262 (66.8%)	<0.001
5 years	621 (79.4%)	286 (73.3%)	335 (85.5%)	<0.001
**Hospital-treated substance-related toxicity in the year prior to death**				
Any	177 (22.6%)	107 (27.4%)	70 (17.9%)	0.001
Benzodiazepine	28 (3.6%)	8 (2.1%)	20 (5.1%)	0.022
Opioid	152 (19.4%)	98 (25.1%)	54 (13.8%)	<0.001
Stimulant	36 (4.6%)	22 (5.6%)	14 (3.6%)	0.167
Alcohol	10 (1.3%)	1−5 (0.3%−1.3%)	1−5 (0.3%−1.3%)	0.057
**Healthcare encounters for substance use disorder**				
Any substance use disorder	501 (64.1%)	259 (66.4%)	242 (61.7%)	0.173
Benzodiazepine use disorder	337 (43.1%)	175 (44.9%)	162 (41.3%)	0.317

† Captures incidents that occurred in collective dwellings, shelters and other residential settings, other indoor spaces (e.g., correctional facilities, hotels/motels/inns, hospitals, transit, etc), and outdoors.

Boldface *p*-value indicates statistically significant difference (α = 0.05) when comparing deaths attributed to approved vs. non-approved benzodiazepines.

## Discussion

In this population-based study of benzodiazepine-related toxicity deaths in Ontario over four and a half years, we observed a low and stable mortality rate prior to the COVID-19 pandemic, followed by a period of elevated rates and increased opioid co-involvement between July 2020 and September 2021, and a subsequent return to pre-pandemic trends by October 2021. Among almost 800 benzodiazepine-related toxicity deaths, we uncovered notable patterns and differences in the characteristics of decedents and circumstances surrounding death when comparing deaths attributed to benzodiazepines approved vs. not-approved for medical use in Canada, advancing the literature on this topic in a Canadian setting. For instance, the higher degree of deaths involving benzodiazepines approved for medical use among older adults and residents of Northern Ontario suggests that regional differences in substance use patterns, as well as varying exposure to contamination of the unregulated drug supply with benzodiazepines are important considerations for public health and harm reduction responses.

Previous investigations of trends in fatal benzodiazepine toxicities have documented an overall increase in the US between 1996 and 2019, with rates peaking in 2017 [19,20]. Across Canada, rates of benzodiazepine toxicity death increased almost four-fold from 1.2 to 5.6 per 100,000 between 2018 and 2023 [9]. Although rates of benzodiazepine-related death were lower in our setting, our findings correspond with reports of increasing presence of benzodiazepines, particularly novel benzodiazepines, in the unregulated drug supply during the pandemic [1,3,21]. The drivers of the increasing adulteration of the unregulated drug supply are complex, and may be related to drug market economics, including rising drug prices and decreasing drug availability leading to the addition of fillers, additives, and other substances in order to reduce costs, [1, 3] as well as the theorized clinical interaction between benzodiazepines and opioids that may amplify the opioid-like effects and increase the length of sedation experienced when these substances are used together [22]. The high degree of exposure to multiple substances observed in our study aligns with findings from Australia, where 97.5% of benzodiazepine-related deaths involved another substance, most commonly opioids [23]. Similarly, among a cohort of people who inject drugs in Scotland, non-prescribed benzodiazepine use was associated with a two-fold increase in non-fatal overdose [24]. These findings are important given that co-exposure of opioids and benzodiazepines complicate the current response to the drug toxicity crisis in several meaningful ways. First, the combined use of benzodiazepines and opioids is known to increase the risk for respiratory depression, [25] making the toxicity response more complex as naloxone cannot reverse the effects of benzodiazepines, thereby heightening the risk of death. Further, frequent exposure to potent benzodiazepines in the unregulated supply can lead to benzodiazepine dependence, leading to withdrawal symptoms, including seizures, upon discontinuation of the unregulated drug supply [26]. Untreated benzodiazepine withdrawal symptoms may also impact retention in opioid agonist treatment therapy when these withdrawal symptoms are inadequately addressed [27–29]. Finally, the risks associated with these novel benzodiazepines have not been well-studied [30]. Taken together, the predominance of opioid involvement in benzodiazepine-related toxicity death observed in our study is reflective of the evolving nature of the drug toxicity crisis, [31,32] highlighting the need for public health and harm reduction responses to be updated to reflect the growing unpredictability of the unregulated drug supply.

The rise that we observed in benzodiazepine-related mortality may also be reflective of pandemic-related changes to the unregulated drug supply, including an increase in novel benzodiazepines contained within the fentanyl supply, [5] pandemic-associated social isolation and mental health impacts, [33] as well as missed opportunities for intervention related to these issues due to service disruptions and closures. Findings from several US jurisdictions indicate that, in addition to increases in mortality attributable novel benzodiazepines during the pandemic, there was also a moderate rise in deaths due to prescription benzodiazepines [34,35]. Although we were unable to examine trends in benzodiazepine deaths by source, it is notable that half of all deaths in our study involved benzodiazepines approved for medical use in Canada. This suggests that the response to the rise in benzodiazepine-related toxicities, and the drug toxicity crisis more broadly, must continue to include efforts to encourage appropriate prescribing and the safe use of prescription medications. However, given concerns surrounding rapid and forced discontinuation of benzodiazepines the benefits and risks of deprescribing in long-term users should be carefully considered with slow tapers recommended [36] given the evidence of increasing risk of suicide, and overdose following discontinuation [37].

Our study also uncovered several notable demographic characteristics and external circumstances associated with benzodiazepine toxicity deaths, as well as key differences in these factors according to benzodiazepine type. Overall, deaths were evenly distributed, with half involving those approved for medical use. Furthermore, in our analysis, more than 95% of benzodiazepine-related deaths, regardless of source, involved a non-benzodiazepine substance, emphasizing the heightened risk for benzodiazepine-related harm when combined with other central nervous system depressants, such as alcohol or opioids. These findings highlight the complexities in the current landscape of substance use and toxicity and demonstrate the need for multifaceted responses to address the different driving factors, including the adaptation of treatment responses to address polysubstance exposure, particularly to benzodiazepines from the unregulated opioid supply. One recent study in the US, [34] also assessed patterns in the characteristics of fatal benzodiazepine-related overdoses. The prevalence of substance co-involvement (opioids, stimulants, and alcohol) and prior benzodiazepine and opioid prescribing reported among decedents was similar to that observed in in our study. The consistency in these results underscores the urgent need for widespread harm reduction efforts, including drug checking services, and the importance of integrating substance use and mental health-related assessments and supports into primary and inpatient care settings.

### Limitations

A core strength of this study is that it is the first to examine trends and patterns in benzodiazepine toxicity deaths using a population-based database in Ontario. However, several limitations warrant discussion. First, in accordance with institutional privacy policy prohibiting the reporting of small cells (counts <5), we were unable to stratify trends in deaths by benzodiazepine type, limiting our ability to understand the degree to which changes in trends were driven by approved vs. non-approved benzodiazepines. Future research should examine how different benzodiazepines may affect toxicity risk, particularly in the context of exposure to multiple substances including fentanyl. Second, our analysis was restricted to deaths in which benzodiazepines directly contributed to the toxicity incident. However, the thresholds for differentiating between whether a benzodiazepine either directly contributed or was involved in death are not well established. Therefore, although our analysis may underestimate the rate of benzodiazepine-related mortality in Ontario, the specificity of our case definition helps to avoid misclassification. Third, information on indication of benzodiazepine use is not readily available in administrative data, and therefore we were not able to assess the reason for prescribing among people whose death arose from a benzodiazepine approved for medical use. Fourth, there is no validated definition of substance use disorders, and therefore we relied on diagnoses made during prior healthcare encounters and receipt of opioid agonist treatment in the prior five years. This definition may under-report people with substance use disorders who are disconnected from the healthcare system, although we anticipate this potential misclassification to be minimal. Finally, we used standard area-level definitions to define neighbourhood income quintile which may only moderately correlate with individual-level income data, and could underestimate the magnitude of sociodemographic differences [38].

## Conclusion

As the unregulated drug supply and toxic drug crisis continues to evolve, it is crucial to continuously monitor trends and patterns in acute toxicity deaths. In Ontario, Canada, we found that benzodiazepines directly contributed to almost 800 toxicity-related deaths over the study period, with nearly all deaths involving multiple substances, most commonly opioids. These findings highlight the growing importance of exposure to multiple substances in the drug toxicity crisis and underscore the need for harm reduction and treatment responses that address both intentional and unintentional benzodiazepine exposure within the increasingly unpredictable unregulated drug supply.

## Supporting information

S1 Table Diagnostic codes and data sources used to define healthcare encounters. (DOCX)
