# Supplementary material for: Characterizing trends and patterns in benzodiazepine-related toxicity deaths in Ontario: A descriptive cross-sectional study
Source: PLoS One. 2026 Sep 15;21(9):e0355210. doi: 10.1371/journal.pone.0355210 (PMC13577399; doi:10.1371/journal.pone.0355210)
Supplement: Supplementary file 1 [file pone.0355210.s001.docx]

**S1 Table.** Diagnostic codes and data sources used to define healthcare encounters.

| **Measure** | **Data Source** | **Definition or Diagnosis Codes** |
| --- | --- | --- |
| **Source of drug contributing to death** | Drug and Drug/Alcohol Related Death Database | **Benzodiazepines not approved for medical use in Canada:** Indicates the presence of benzodiazepines with primarily unregulated origins (i.e., etizolam, flubromazolam, flualprazolam, and bromazolam).  **Benzodiazepines approved for medical use in Canada:** Indicates the presence of benzodiazepines with regulated origins (i.e., alprazolam, bromazepam, camazepam, chlordiazepoxide, clobazam, clonazepam, demoxepam, diazepam, flurazepam, hydroxyalprazolam, lorazepam, midazolam, nitrazepam, oxazepam, temazepam, and clorazepate). This category may include benzodiazepines that were prescribed to the deceased person or that were prescribed to someone else (i.e., diverted).  **Opioids from the unregulated supply:** Heroin, heroin metabolites (morphine where monoacetylmorphine (6-MAM) was also detected), U-47700, fentanyl, fentanyl analogues (including carfentanil).  **Pharmaceutical opioids:** Buprenorphine, codeine, hydrocodone, hydromorphone, methadone, morphine where 6-MAM was not detected, oxycodone, oxymorphone or tramadol. This category may include opioids that were prescribed to the deceased person or that were prescribed to someone else (i.e., diverted). |
| **Substance involvement in opioid toxicity deaths** | Drug and Drug/Alcohol Related Death Database | **Directly contributing to death:** Substances determined by the pathologist and/or coroner to have directly contributed to the death based on the complete investigative findings (i.e., toxicology findings and the information obtained during the death investigation).  **Involved or detected in death:** Substances detected in toxicology testing, which may or may not have directly contributed to the death. |
| **Healthcare encounters for acute substance-related toxicities *(past 1 year)*** | National Ambulatory Care Reporting System (all diagnosis types), Discharge Abstract Database (admission diagnosis) | **Benzodiazepines:** ICD-10 code T42.4  **Opioids:** ICD-10 codes T40.0, T40.1, T40.2, T40.3, T40.4, T40.6  **Alcohol:** ICD-10 code T51.0  **Stimulants:** ICD-10 codes T43.6, T40.5  **Any Substance:** A healthcare encounter for any of the above substance-specific toxicities |
| **Healthcare encounters where substance use disorder diagnosis was recorded**  ***(past 5 years for emergency department visits and inpatient hospitalizations, past 1 year for outpatient visits)*** | OHIP Claims Database, National Ambulatory Care Reporting System, Discharge Abstract Database, Ontario Mental Health Reporting System | **Benzodiazepine Use Disorder**   - ***NACRS/DAD/OMHRS (past 5 years)****:* ICD-10 code F13; ICD-9 code (OMHRS only) 304.1   **Opioid Use Disorder**   - ***NACRS/DAD/OMHRS (past 5 years):*** ICD-10 code F11; ICD-9 codes (OMHRS only) 304.0, 305.5 - ***OHIP (past 1 year):*** Fee (billing) codes K682, K683 & K684   **Alcohol Use Disorder**   - ***NACRS/DAD/OMHRS (past 5 years):*** ICD-10 codes F10, K70, G312, G621, G721, I426, K292, K860, Z502, Z714, Z8640, E244, K852; ICD-9 codes (OMHRS only) 291.x [excl. 291.82], 303.x, 305.0 - ***OHIP (past 1 year):*** OHIP dxcode 303, 291   **Stimulant Use Disorder**   - ***NACRS/DAD/OMHRS (past 5 years):*** ICD-10 codes F14, F15; ICD-9 code (OMHRS only) 304.4   **Any Substance Use Disorder:**  Any healthcare encounter where a substance use disorder was recorded was defined as any hospital diagnosis in the last 5 years with any of the above substance use disorders/alcohol use diagnosis codes recorded, or any outpatient visits in the past year with any of the above substance use disorder/alcohol use disorder diagnosis codes recorded |

**Abbreviations:** DAD – Discharge Abstract Database; DSM – Diagnostic and Statistical Manual of Mental Disorders; ICD-10 – International Classification of Diseases, 10th Revision; NACRS – National Ambulatory Care Reporting System; OHIP – Ontario Health Insurance Plan; OMHRS – Ontario Mental Health Reporting System
